# Patient informed consent, ethical and legal considerations in the context of digital vulnerability with smart, cardiac implantable electronic devices

**DOI:** 10.1371/journal.pdig.0000507

**Published:** 2024-05-23

**Authors:** Leanne N. S. Torgersen, Stefan M. Schulz, Ricardo G. Lugo, Stefan Sütterlin

**Affiliations:** 1 Department of Behavioural Medicine and Principles of Human Biology for the Health Sciences, Trier University, Germany; 2 Estonian Maritime Academy, Tallinn University of Technology, Estonia; 3 Faculty of Computer Science, Albstadt-Sigmaringen University, Germany; University of Washington, UNITED STATES

## Abstract

Advancements in digitalisation with cardiac implantable electronic devices (CIEDs) allow patients opportunities for improved autonomy, quality of life, and a potential increase in life expectancy. However, with the digital and functional practicalities of CIEDs, there exists also cyber safety issues with transferring wireless information. If a digital network were to be hacked, a CIED patient could experience both the loss of sensitive data and the loss of functional control of the CIED due to an unwelcome party. Moreover, if a CIED patient were to become victim of a cyber attack, which resulted in a serious or lethal event, and if this information were to become public, the trust in healthcare would be impacted and legal consequences could result. A cyber attack therefore poses not only a direct threat to the patient’s health but also the confidentiality, integrity, and availability of the CIED, and these cyber threats could be considered “patient-targeted threats.” Informed consent is a key component of ethical care, legally concordant practice, and promoting patient-as-partner therapeutic relationships [1]. To date, there are no standardised guidelines for listing cybersecurity risks within the informed consent or for discussing them during the consent process. Providers are responsible for adhering to the ethical principles of autonomy, beneficence, non-maleficence, and justice, both in medical practice generally and the informed consent process specifically. At present, the decision to include cybersecurity risks is mainly left to the provider’s discretion, who may also have limited cyber risk information. Without effective and in-depth communication about all possible cybersecurity risks during the consent process, CIED patients can be left unaware of the privacy and physical risks they possess by carrying such a device. Therefore, cyber risk factors should be covered within the patients’ informed consent and reviewed on an ongoing basis as new risk information becomes available. By including cyber risk information in the informed consent process, patients are given the autonomy to make the best-informed decision.

## Author summary

Cardiac implantable electronic devices (CIEDs) allow patients opportunities for improved autonomy and quality of life. However, CIEDs possess cyber safety issues and patients may not be aware of these risks. As there has not been a case made public yet of a patient with a CIED becoming victim to a cyber attack, one could rationalise these cyber attacks as presently being only speculative in nature. However, the chance of such an attack occurring is increasing as these devices possess known cyber vulnerabilities, which have been published for over a decade. In addition, to date, there are no standardised guidelines for identifying cybersecurity risks within the informed consent or for discussing them during the consent process. With these safety concerns, we theorise: (1) Cyber risk scenarios are difficult to plan for as they constitute an “ambiguous threat” and result in ineffective protective measures; (2) cyber threats can impair trust in the device, the treatment plan and the patient provider relationship; and (3) the perceived threat to CIED patients is elevated because CIEDs are mobile with the patient, the threat can not be quantified, and the cyber risk stress to the patient can be higher than from a technical malfunction. Institutions and providers that have not informed their CIED patients about cybersecurity risk(s) are implicitly altering patients’ risk perceptions and treatment choices. From conducting a literature search and review, we discuss our findings and provide suggestions to the ever-increasing safety challenges with cybersecurity and the emerging “patient-targeted threat” to CIED patients. We assess the ethical implications and propose solutions for continued adherence to patient-centered care (PCC) practices and for validation of the informed consent process and content.

## Introduction

### Digitalisation of implantable medical devices

With advances in digitalisation of cardiac implantable electronic technologies, patients have greater opportunities for improved autonomy, quality of life, and possible increase in life expectancy. Information technology has improved the coordination between mobile apps, medical devices, and the healthcare provider or electronic medical database for both the delivery and exchange of needed medical data or information. This streamlining of information, with an implantable medical device in a patient, allows for the reduction of face-to-face interactions, surgical procedures, or routine maintenance visits, thus supporting cost-effective practice measures. In the case of cardiac patients with cardiac implantable electronic devices (CIEDs), current models are capable of: (1) monitoring physiological function in real-time by acquiring data automatically on a frequent basis (e.g., daily); (2) alerting a provider to any information and warnings about device integrity; and (3) allowing for remote monitoring of the patient’s health condition without any manual interventions from providers [2,3]. For patients with significant cardiac disease and/or arrhythmias, implantable electronic devices such as permanent pacemakers, defibrillators, and cardiac resynchronization therapy (CRT) devices can assist with maintaining baseline cardiac function for the patient and the avoidance of life-threatening outcomes [4]. Sensors within the devices can transmit patient data such as respiratory rate (RR), physical activity levels (based on vital sign values), short recordings of intracardial cardiograms, heart rate (HR), night-time pulse, temperature, a rapid-shallow-breathing-index, thoracic impedance, and heart-sounds [5]. In addition, there is an algorithm within the devices that can use multiple sensors to track trends and alert providers if worsening heart conditions are identified in the patient [3]. Thus, implantable cardiac medical devices can provide instantaneous communication to the provider of the patient’s functional, cardiac status and can prompt follow-up procedures if needed, including: (1) rapid treatment and response if the patient’s status deteriorates (such as the delivery of a life-saving electrical shock due to a life-threatening arrhythmia); and (2) reassurance that there is immediate support should a serious event occur, as the device communicates directly to the provider in real-time. As a result, CIEDs are utilised increasingly to support care remotely and provide a means for physicians to adjust therapy without requiring an office visit [6]. Web-based support systems (e.g., Schulz et al., and Pedersen et al. [7,8]) offer means for an integrated information management of personal and medical data including online monitoring of CIED readout. This is highly attractive for providing optimised interprofessional support for CIED carriers. However, this adds an additional layer of security vulnerabilities that apply to any web-based application that is part of the world-wide-web [7,8].

### Vulnerability of smart CIEDs

The wireless transfer of information requires interfaces that come also with inherent security risks. Since most devices contain proprietary communication protocols that do not allow direct access to the internet, they connect instead to the internet via a secondary device, such as a bedside monitor or a mobile device [9]. In addition, some CIEDs release medical applications using Bluetooth technology, which often utilises software-defined radios so a device can operate over multiple frequencies, while others utilise Wi-Fi, Cellular, and Ethernet connectivity options for transferring data to external manufacturer servers and eventually on to the provider [9–11]. As a result, the Internet of Things now incorporates the Internet of Medical Things with the inclusion of the implantable electronic device network since this network digitally connects patients to providers and transfers both sensitive data and operational device details. However, with the optimisation of medical communication, there exists a significant risk to patients who possess CIEDs especially if the digital network were to become compromised. A single error in the implementation of a security protocol or access vulnerability could allow for an unchecked code to run and permit the opening or modification of the entire cyber system [12]. Thus, through a single weak entry point, a cyber breach has the potential to affect both the health care institution and the individual patient [13]. Possible CIED patient outcomes resulting from a network compromise could include both the loss of sensitive data as well as the loss of control of the CIED to an unknown third party, which would not only impact the integrity of the CIED but can also pose direct threats to the patient’s privacy and personal health. Thus, for those with CIEDs, this cybersecurity threat is realistically a “patient-targeted threat.” Compromised digital networks are a common and fast-growing societal problem and the healthcare sector is particularly prone to be targeted by cyber attacks (see, e.g., Sütterlin et al. [14]). In the specific case of CIEDs, the Food and Drug Administration (FDA) has issued safety communications directed towards providers, caregivers, and patients warning of needed upgrades in device technology. For example, the FDA issued a warning letter to Abbott Laboratories in 2016, after a report by Muddy Waters LLC outlined 2 methods, the “crash attack” and the “battery drain attack,” that could be implemented and thus directly impact the patient containing the CIED [15].

With the ever-increasing number of cyber attacks and cybercrimes occurring, the possibility of having complete immunity from such attacks diminishes. According to Nifakos et al. [16], “94% of healthcare organisations globally have experienced data breaches of patient records, encountered information loss, been hacked or had their data displaced.” Thus, not all of the Internet of Medical Things’ technologies and protective algorithms are cyber resilient enough to withstand the advanced sophistication of cyber attacks being implemented. Once the unavoidable and technology-inherent vulnerabilities become further known, cyber hackers and criminals with commercial interests and knowledge about these digital vulnerabilities will easily exploit and sell accessed data on the markets on the dark web. Cyber hackers have advanced their techniques in breaking through security protection so efficiently and effectively that conventional cybersecurity techniques are no longer able to detect “zero-day attacks” [17]. Thus, this potential exploitation underlines the necessity to prepare healthcare systems and their processes for an age where security breaches can and will affect patients’ lives.

**Fig 1:**
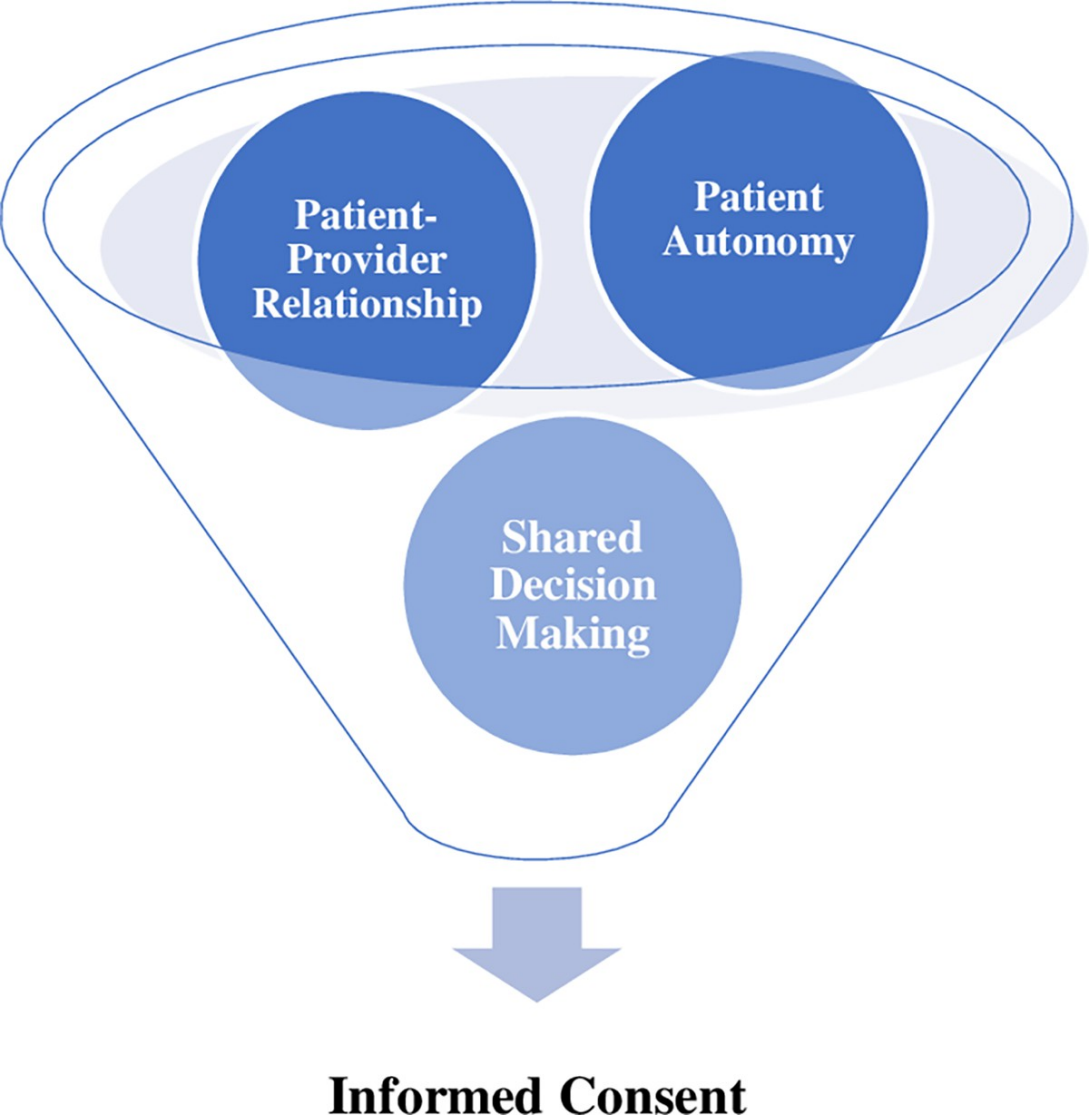
Conceptual illustration of key components of informed consent. Conceptual illustration created by Leanne Torgersen using SmartArt Graphics in PowerPoint, June 2023.

Technological vulnerabilities threaten the confidentiality, integrity, and availability of the digital network and the healthcare services provided as well as the trust in the relationships between the patient–provider and the patient–medical institution. There has not been a case made public yet of a patient with a CIED becoming victim to a cyber attack. Hence, one might consider these threats hypothetical until such an attack has been made public. However, the chance of such an attack occurring is increasing, and as reported above, proof of principle has been accomplished under realistic conditions. When considering the impact of IT vulnerabilities with CIEDs and the implications to medical practice and patient safety, we postulate the following premises on which we build our case: (1) **Low level of institutional readiness:** Low-probability high-impact scenarios are difficult to imagine or plan for (as in representative heuristic, see e.g., Das et al. [18]) and constitute an “ambiguous threat.” Ambiguous threats are associated with delayed planning as well as ineffective and inconsistent implementation of protective measures. In most cases, these threats are often downplayed or ignored. Research on publicly perceived security suggests that once a case of CIED-hacking is made public, will the perceived patient threat level increase and only then will it influence decision-making rather than the latter having commenced prospectively; (2) **Trust:** These threats impair patients’ trust in the device, in the treatment plan, and in the relationship with the provider and/or medical institution; of note, once a sufficiently dramatic successful hacker attack is reported in the media, trust in CIEDs will be undermined for an extended period of time, and may be generalised to similar devices as suggested by research on the effects of device malfunction and recalled, e.g., due to production issues [19]; and (3) **Subjective threat potential:** Compared to cyber threats in the healthcare sector, where medical devices are temporarily out of order as a result of a cyber attack, the perceived threat (as depicted under point 2 above) is increased in smart CIEDs due to: (1) being unlike other medical devices, the CIED is not stationary but travels with the patient, rendering the perceived threat a consistent “patient-targeted threat” with concomitant and additional consequences of mental injury; (2) the threat can not be quantified in a usable way as it is not statistical; and (3) being subjected to a crime, including the malicious intent of a third party, can cause more stress than a purely technical failure with the device itself. Points (1) to (3) indicate that those institutions that are failing to prepare their patients for the risks of CIED-reliant treatment in the presence of this highly individualised, inescapable, and unquantifiable vulnerability, have the potential to unwittingly shift patients’ risk perceptions and thus treatment preferences and choices such as consenting to an implantable device. These adjustments in health care decisions could also be potentially mediated by public coverage of first incidents and resulting public debates and (mis)perceptions. Based on these combined observations, we propose that a treatment risk factor such as a potential security breach should—like other potential treatment risks—be covered by the patients’ informed consent and reviewed throughout the treatment process when new risk information becomes available. As the patient has a legal and moral right to be informed of the risk prospectively and not retrospectively, this has implications for the informed consent process [20]. Informed consent is a key component of both ethical care and promoting therapeutic relationships through chosen patient-centered treatment plan(s) (see Fig 1).

The practice of informed consent is shaped by societal, ethical, and most of all legal frameworks defining providers’ obligations and liabilities. The process of informed consent requires accurate, authentic, and reliable communication between the provider and patient. In addition, providers may not be necessarily prepared to inform patients about cybersecurity risks and/or they may not have all the information themselves. As described, the current requirements of the informed consent process and shared medical decision-making practices are meant to empower patients in these processes. With the increase in cybersecurity risks, challenges remain as to how healthcare systems can continue to adhere to their legal and ethical duties if these cyber risks are not addressed in the process of informed consent. The aim of this review is to bring into focus the increasing and known cyber vulnerabilities with CIEDs, to propose solutions for continued adherence to patient-centered care (PCC) practices, and to suggest incorporating foreseeable cyber risk information in the informed consent. Thus, this review highlights the ever-growing “patient-targeted threat” to those patients with CIEDs and the increased need for cyber risk transparency in the informed consent and consenting process. Finally, this review examines how PCC will need to evolve and adapt to these known and increasing cybersecurity risks.

### Patient-centered care

“Patient-centeredness understands that population-derived, scientific knowledge requires translation to each individual’s unique situation, paralleling the distinction between public health and medicine, and acknowledges with humility that not only do we have insufficient scientific knowledge for purely evidence-based practice that is constrained by guidelines, but that such a reductive, technocratic approach is counter-productive when working with real, whole people” [21]. Moreover, PCC focuses on care from a biopsychosocial perspective, which combines psychotherapeutic theories in encouraging the patient to disclose his/her real concerns, to empower the patient with negotiating, and to support the patient in deciding on a care plan equally with the provider [22]. PCC has developed over time both from the advancements in technology as well as the expansion of the fundamental concepts of patient choice, shared decision-making, patient–provider relationship, patient education, and increased access to information via digital solutions. Regardless, the focus of PCC emphasises: (1) what is best for that particular person/patient; (2) what care or treatment will provide the best outcome physically, mentally, and spiritually for that individual; and (3) will that individual be satisfied overall with one’s life trajectory based on that individual’s own personal values. According to Stewart [23], PCC is described as care that: (1) explores the patient’s reason(s) for his/her health visit and concerns; (2) supports medical staff developing a holistic understanding of the patient’s social, emotional, and physiological viewpoints; (3) allows for open dialogues between medical staff and the patient about the health concern in question and the shared management of it; (4) promotes empowerment of the patient both in his/her care as well as overall happiness; and (5) encourages a continuing and strong relationship between the patient and the health care provider. Along with digitalisation in the medical field, healthcare in industrialised countries is continually evolving with its practice of patient-centered healthcare, and in collaboration with the provider, emphasises shared decision-making with the health services chosen that were tailored to the individual’s needs, preferences, rights, autonomy, and values.

The emergence of cybersecurity risks, such as “being hacked” by anonymous individuals with malicious intent, can shape and alter how we view and practice PCC, what risks are communicated on the informed consent and how to maintain the patient’s ability to make an informed decision. We conducted a literature review using the following search engines: PubMed, Cochrane Libraries, Scopus and ScienceDirect, Google Scholar, and ResearchGate. Methods of literature searching included the systematic search method using combinations of the key words “cardiac implantable electronic devices,” “informed consent,” “cybersecurity,” “cyber risks,” and “ethics” and the snowball method for author/literature searching. From conducting this literature search and review, we found that there were minimal discussions or dialogues with patients regarding potential risks and there was no general practice or consensus among hospitals and companies on how to implement emergency measures when a breach has occurred. In addition, hospitals, who primarily purchase the CIEDs from manufacturers, receive limited information on device details and performance, which in turn is needed for providers to convey pertinent information to patients who are considering obtaining a CIED or who already possess one [24]. Thus, how well both physical and privacy-related risks associated with cyber attacks are presented to the patient during the informed consent process is unknown, which suggests that what is actually communicated to the patient is provider and device company dependent. This implies that some may provide less detail without recognising the potential serious risks and consequences. As the threat of CIEDs being compromised is ever-growing, whether directly or indirectly through attacks to hospital or company systems, the adequacy, quality, and transparency of communication between companies, providers/hospital staff, and especially the patient needs to increase. In addition, the challenge remains as to how to communicate these risk(s) accurately and in terms that can be understood by the patient. With regard to how to address and describe cybersecurity threats to patients, there is not a shortage of information, but rather an overwhelming amount of it, which adds to the already enormous complexity for patients who try to make an informed decision [14]. Given the abundance of information on the numerous cyber risks with CIEDs, such risks need to be conveyed, in a commensurate manner to the possible toxicities of a medication, so that the information is presented in a standardised, regulated, and evidence-based fashion rather than a subset of risk-related information being decided and/or downplayed by other involved parties and/or influenced or exaggerated by media coverage.

## The healthcare sector’s cyber vulnerability and how it impacts patients with CIEDs

**Fig 2:**
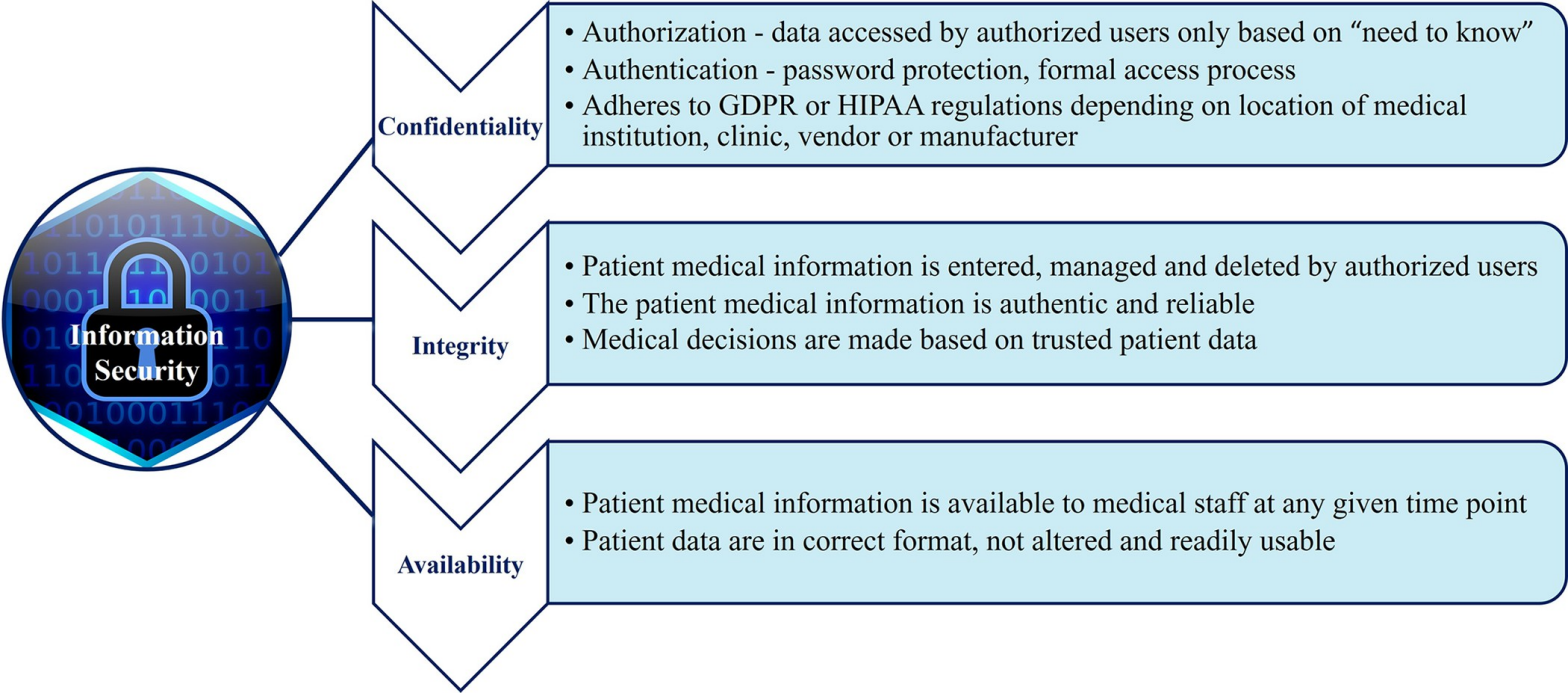
The cybersecurity triad for protecting patient medical information, defined by NIST and NCCoE (December 2020). GDPR, General Data Protection Regulation; HIPAA, Health Insurance Portability and Accountability Act; NIST, National Institute of Standards and Technology; NCCoE, National Cybersecurity Center of Excellence. Image for information security contributed by citiany and downloaded from CleanPNG, April 2024. Caption credit: Cawthra, J, Ekstrom, M, Lusty, L, Sexton, J, Sweetnam, J, Townsend, A. NIST Special Publication Data Integrity: Detecting and Responding to Ransomware and Other Destructive Events. Available from: Executive Summary—NIST SP 1800–26 documentation, downloaded 10 June 2023.

In general, there are 3 pillars of information security and cyber compromises to it can affect the confidentiality of data within it, the integrity or trust in the digital network, and data that is tasked to protect and the availability of the data it contains [25] (see Fig 2).

**Fig 3:**
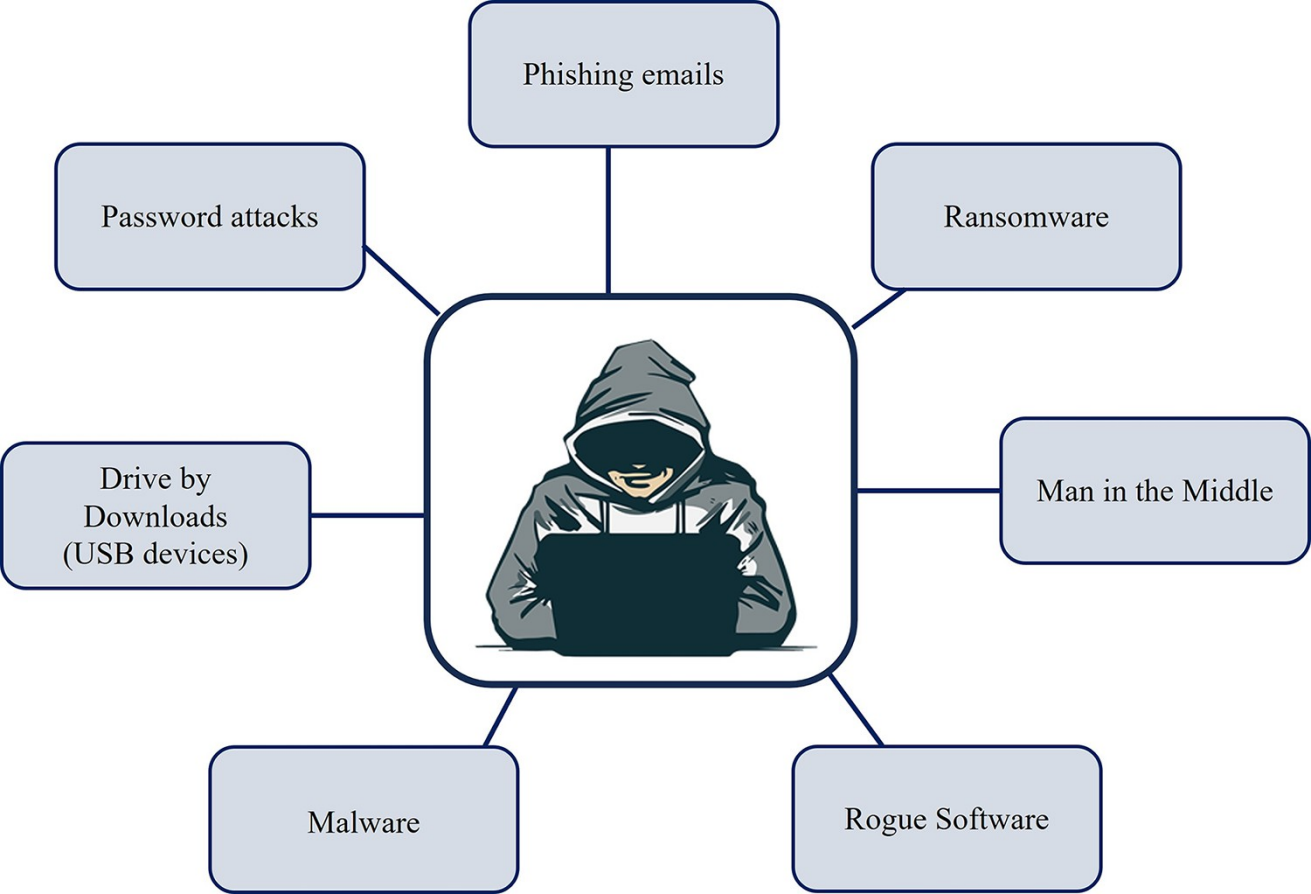
Conceptual illustration of methods hackers utilise for infiltrating medical institutions and/or medical devices. Image of hacker contributed by kanila and downloaded from CleanPNG, April 2024.

These pillars of information security are based on the operational processes that are in place to protect the data it contains whether at a medical institution, an outpatient clinic, or external manufacturer programmer. Operational cybersecurity risks are formally defined as the “operational risks to information and technology assets that have consequences affecting the confidentiality, availability, and integrity of information or information systems” [26,27]. As cybersecurity rests upon these 3 main pillars of operational data protection: confidentiality, availability, and integrity, each of these pillars can affect patients with CIEDs whether physiologically and/or through the invasion of privacy [28,29]. Even if these operational data systems are well maintained or resilient, there are opportunities whether through the technology, the protocols or process or the people, that a cyber attack can still penetrate a data network. Thus, these digital networks and their respective cybersecurity protocols can have both direct and indirect effects to the medical institution’s operational processes, the patients, and the patient services provided should they become unlawfully accessed (see Fig 3).

Cyber attacks, targeting breaches in confidentiality, focus on exposing private information (such as health information through patient medical records) to unauthorised persons. Implantable electronic device data listed in a patient’s medical record as well as within the implanted device itself can be made available and sold to others without the patient being aware that their information has been stolen. The operational data integrity pillar represents the measures taken to prevent an unauthorised party from modifying data or a network system, which can also include altering data on medical devices. Attacks against data integrity modify the data or the device itself in such a way that it becomes untrustworthy, and the attack may not be discovered until after the damage has been done. An example of a data integrity attack includes a “man in the middle” type of attack [6], where a hacker enters the medical records within a hospital, modifies the data within its network, and presents the information as if it were authentic. Unsuspecting medical personnel and providers could therefore make changes to treatment plans, such as modifying the medication dosage or the settings or programming of the CIED without realising that the data were not accurate. Jay Radcliffe, a cybersecurity researcher and diabetic, was able to hack into his own implanted insulin pump device and adjust the insulin settings to show how feasible and easily accessible it was to impact the integrity of the device [30,31]. Jack Barnaby, who is also a diabetic, took it one step further and demonstrated on a mannequin his ability to hack the insulin pump and inject a lethal dose of insulin into the mannequin’s pancreas [30]. Of particular concern is how these examples of cyber attacks could be easily applied to CIEDs. Finally, the availability of data focuses on information assurance, which ensures that authorised users are able to access the data when needed. Examples of availability attacks include the multitude of ransomware attacks against hospitals and healthcare facilities that have been observed over recent years [2] (see Table 1).

**Table 1:** Possible negative outcomes to medical institutions and CIED patients from successful cyber attacks.

Examples of outcomes	Cybersecurity Triad		
	Confidentiality	Integrity	Availability
1	Data breach, stealing of patient medical information and sensitive data, selling on the dark web	Man in the Middle: manipulating or intercepting data	Ransomware—interrupting or blocking access to data
2	Cracking data encryption or installing unauthorised encrypted spyware	Accessing servers—stealing, illegally altering or deleting data or interrupting of blocking data transmission	Denial of service (DoS), Distributed denial of service (DDoS)—disrupts availability of data
3	Installing malware or spyware on the server	Resource depletion, such as battery drain to a CIED	Malware disrupting or flooding server—limits or blocks access to patient data
4	Identifying location of CIED to other hackers	Erroneous defibrillation administration	Medjacking medical machines—altering or blocking use

Caption credit: Das, S, Siroky, GP, Lee, S, Mehta, D, Suri, R. Cybersecurity: The need for data and patient safety with cardiac implantable electronic devices. Heart Rhythm. 2021;18(3):473–481. https://doi.org/10.1016/j.hrthm.2020.10.009.

In 2015, a Russian cyber group, known as Sandworm, unleashed malware called NotPetya that “knocked out” both hospitals and pharmaceutical manufacturers simultaneously, which limited the availability of patient data for effective patient care management [32]. A hacker could in essence halt the entire electronic medical record system of a hospital especially if the institution had not effectively implemented a reliable data backup system to ward off the consequences of such an attack. This type of cybersecurity risk could again leave the patient with the implantable device unaware of the lack of oversight or monitoring of his/her device and health status. If a medical emergency were to occur to the patient during this period, valuable medical information may not be readily available, and this could result in a potentially lethal outcome to the patient. As a result, patients with CIEDs “carry” an extra risk associated with having such a device, and thereby increase their risk for personal attacks based on interpreted personal gain for hackers (e.g., financial gain, fame, national security interests) [33].

For some medical institutions, one barrier to prioritising cyber-resilience is the cost of investing and managing in something that takes resources from the institution’s primary role [34]. As a result, the level of effort for cyber protection is generally based on the funding available, which can vary with each institution as well as the country in which it is located. Moreover, “there is a critical lack of national audits within the healthcare industry, which reports on cyber resilience” [16]. A related barrier with implantable devices is that there is still no set standardisation of guidelines on how to address the security and privacy issues associated with these devices despite awareness of the issue for over a decade [33]. In addition, perpetrators in most cases can not be clearly identified (referred to as the “attribution problem”), this can add an additional component of insecurity for the victims of cyber attacks [14]. As cybersecurity attacks remain ambiguous in nature, some medical institutions, due to their financial constraints, may instead choose to downplay and/or ignore these threats resulting in a continued disjointedness to the execution of cyber-resilient practices and protection that could affect patients with implantable devices. Based on the existing ineffective or absent cyber protection practices against technological and human vulnerabilities, the risk of cyber attacks will continue to increase with the ongoing digitalisation and the potential opportunities to exploit it.

## The risks to patients with cardiac implantable electronic devices (CIEDs)

**Fig 4:**
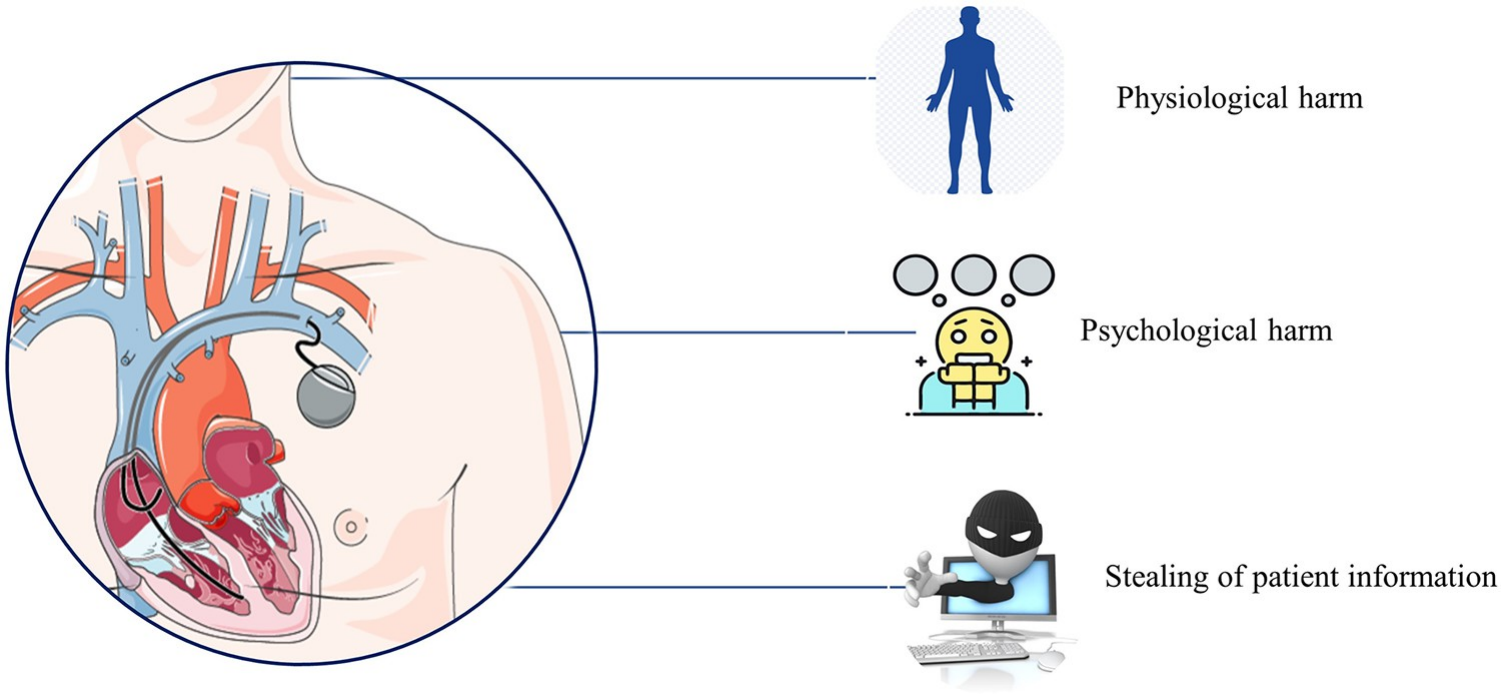
Conceptual illustration of the 3 distinct but interlocking risks to CIED patients. Image for pacemaker created by unknown author and downloaded from Smart Servier Medical Art (under license CC-BY 4.0). Images for physiological harm, psychological harm, and stealing of patient information created by lynndell, yanai, and ramanibhai, respectively, and downloaded from CleanPNG, April 2024.

For patients with CIEDs, there exist 3 distinct but interlocking risks, which are the actual and legal risks for physical harm, for mental harm, and for breach of privacy/confidentiality (see Fig 4).

Altering a CIED with the intent to cause harm to a patient can include mechanistic approaches such as: battery depletion, deactivating or altering features to trigger an emergency when the patient is actually stable; delaying, interfering or blocking communications; and interfering with electromagnetic frequencies [35]. A CIED could be accessed and forced to remain continually engaged by an unauthenticated device or wireless communication, which could cause either the battery life to be consumed faster, potentially leading to malfunction or the implementation of “denial-of-service” and blocking access to needed data on medical networks/servers [36]. If there were an actual medical emergency occurring to a patient with a CIED, the network may not be aware of the emergency as the device may not be functioning effectively or able to communicate properly between the patient and provider/healthcare team. Similarly, a hacker could also manipulate the data on a pacemaker, thereby administering a potentially lethal, electrical shock to the patient when one was not medically required [37]. In comparison, electromagnetic interference to a CIED could cause either: (1) the detection of non-physiological signals that could deliver an inappropriate shock or inhibit pacing or device functioning; or (2) the spontaneous reprogramming of the device [38]. In 2008, a team of researchers from Harvard University, the University of Massachusetts Amherst, and the University of Washington demonstrated, by using an off-the-shelf radio and some computer equipment, how hackers could easily obtain patients’ private medical information as well as reprogram the CIED to not only administer a fatal shock but also shut down its stored settings rendering it non-functional in an emergency situation [9]. In addition, Halperin et al. [36] discussed the need for improved security for cyber attacks after they conducted several software radio-based cyber attacks to an implantable cardiac defibrillator (ICD) through reverse-engineering using an oscilloscope and software radio. These types of attacks can lead to both false positive and false negative conclusions about the patient’s health status and all of these physical risks can have lethal outcomes to the patient [33]. Another concern is the breach of privacy with a patient’s identifiable information and medical condition(s). CIEDs maintain personal and sensitive information about the patient such as vital signs, diagnosed conditions, therapies as well as personal data (name, date of birth, and other pertinent identifiers) [39]. In addition to private information being stored on the device, there is an increased access to the patient’s sensitive data as it is bundled, stored, and processed in centralised or highly interconnected digital infrastructures based on the permitted agreement and cooperation between a given patient and numerous other actors in the healthcare system [14]. Finally, universal serial bus (USB) devices, which are typically utilised by the provider and health staff to access, print or transfer needed patient data for the monitoring of the patient’s status as well as the device’s outputs and function can be yet another weak point for hacker entry. Hackers could obtain unauthorised access through the USB port by injecting malicious malware whether through an external programmer, a provider database, or a home monitor, and possibly read the stored data and adjust the settings to the device [18]. As a result, patients with CIEDs are incurring two potentially profound, consequential, and direct risks due to the multitude of cyber attack options. The first risk is patients could experience lethal outcomes due to unwanted alterations to their devices or from disrupted communications with providers and healthcare networks. The second risk is patients could also be subject to confidentiality attacks with the potential selling of their personal information on the black market. The concern at present is how much patients, with CIEDs or those considering obtaining one, are aware of these cyber risks and the possible serious adverse events from them. Both of these risks carry with them an indirect psychological risk of causing distress to the patient both in the concern of “carrying the risk at all times,” but also the psychological risk should an attack actually occur and the outcome from it.

## Ethical considerations with the level of truth disclosed

With regard to the dialogue between the patient and provider, countries can have different cultural values on what should be communicated to the patient and how much detail should be provided during the consent process or in dialogue together. For some providers, there is concern that more stringent disclosure requirements would risk: (1) overwhelming patients with information, causing distress or leading patients to make poor decisions; and (2) encroaching on the provider’s time due to the possible need in providing more detailed and extended explanations [40]. Typically in the United States, full disclosure to the patient, despite how grave the disease diagnosis or prognosis is, is now the norm [41]. However, the US courts in general agree with non-disclosure if the medical information would pose a threat or cause psychological harm to the patient resulting in the patient’s inability to make a rational decision due to becoming emotionally distraught [42]. In the case of CIEDs, some providers might argue that if a patient determines the risk of serious injury from a cyber attack outweighs the benefit of the device, then the provider may feel he/she has the right to withhold the information as it was done in the patient’s best interest and if there were no other viable options of treatment available. While withholding risk information may be reflective of culture and legal practices within specific countries, how much is truly being conveyed to the patient about his/her cyber risk by possessing such a device remains unknown and again, should a cyber attack be effective in causing a serious adverse outcome to a patient, this could be costly in reputation to the provider. In the US, a provider cannot withhold information simply due to the belief that the patient may refuse a specific treatment and therefore must disclose the information that a reasonable person would want to have for his/her personal decision-making, even if that information may cause the patient to refuse the treatment despite the provider’s medical opinion that the treatment is in the patient’s best interest [42]. Also, due to the litigation tendencies and practices in the US, this could lead to a possible increase in the number of “negligent nondisclosure” legal cases especially if patients and their families perceive that the injury and/or stealing of private information was directly connected to a cyber breach to a CIED and these cyber risks had not been disclosed beforehand. Regardless of country and cultural practice, due to the rise of cyber attacks on all critical infrastructure sectors, patients should be provided all foreseeable risks and benefits of obtaining a CIED and based on their values, interests, and beliefs, be able to make the most informed decision about their health and choice of treatment regardless of the opinions of others. “Without patient-centeredness, medicine can lose its human face and leave the patient alone amidst the medical technology.” [22]. It is the ethical principle of autonomy in the informed consent process that is pertinent to maintain and honour in the end for it is the patient who shall incur the “patient-targeted threat” and will be affected by both the benefits and risks of a treatment or device.

In comparison to the US’s growing standardisation of increased transparency between the patient, family, and provider, full disclosure to patients and/families can differ among other countries [43]. One landmark legal case, Montgomery vs. Lanarkshire Health Board [2015] UKSC 11 [44], brought about a shift in Scotland from a paternalistic medical opinion dominance with the informed consent process to focusing on the patient’s rights to be informed of the risks with a treatment and to decide on which treatment is best for the patient after knowing the risk and treatment alternatives (autonomy to decide) [45]. In this particular case, a diabetic woman delivered a larger sized baby due to her preexisting condition and was not informed of the risks nor were other treatment options discussed due to the provider’s assessment that the risk of serious injury to the baby from the birthing process would be small [40,45]. Consequently, the newborn suffered serious complications from the delivery, which resulted in a permanent change in physiological baseline and perceived quality of life for the child. From the initial ruling, the medical profession was granted the right to withhold information if: (1) the provider deemed disclosing would be mentally distressing or detrimental to the patient’s health; and (2) the patient would have refused the alternate treatment even if it had been disclosed [46]. This suggested that the concepts of patient-participation and empowerment were often challenging for providers to grasp in the larger concepts of patient-centeredness and the patient’s right to choose [21]. However, the case was appealed and went to the Supreme Court, which overturned the previous ruling, thereby setting a new standard for informing patients about medical procedures. Some of key decisions from the Supreme Court ruling highlighted: (1) the need for an adult of sound mind to decide; (2) the provider’s role to ensure a patient is informed of the benefits and any material risks with the treatment as well as any reasonable alternatives; (3) the provider should be aware that each patient will place a level of significance to each risk and therefore the risk assessment is both fact-sensitive and sensitive to the characteristics of the patient; and (4) the avoidance of dialogues that are perceived to cause harm to the patient should not be abused [44–46]. Personalization of health care services and patient’s autonomy do not contradict nor should be thought of as a challenge against evidence-based practice, but rather acknowledges and embraces the patient’s needs as an individual when choices are being made for that individual’s condition [21]. When professional providers select what information to disclose (should they be in receipt of such information and yet determine it is not of needed value or would provide greater unnecessary concern/anxiety to the patient), this withholding of information is in direct opposition to the ethical principle of respect for persons/autonomy. Thus, transparency with disclosing all risk information and treatment options during the consent process will not only ensure shared decision-making is practiced, but it is crucial for supporting patient autonomy when deciding lifesaving but potentially high-risk treatment options such as CIEDs.

## How patient-centered care and the informed consent process need to account for cybersecurity risks

With the increasing complexity of cybersecurity threats targeting hospitals and patients with CIEDs, both PCC practices and the informed consent process will need to adapt. As described by Sütterlin et al. [14], the challenge with consents is to provide an accurate yet simplified description(s) of what possible cybersecurity threats exist to those patients who are either considering an implantable electronic device or currently possess one. Most informed consents, on average, are standardly written at a sixth grade reading level so descriptions of risk may need to be adjusted accordingly [47]. Thus, communicating possible cyber risks at a language level (e.g., layman’s terms), which allows for patients to comprehend the information, adds to the further challenges with the consent process especially since science, technology, and genetic personalization are constantly advancing [48].

When factoring key patient-centered practices such as shared decision-making and patient empowerment as well as the ethical principles of respect for persons/autonomy, justice, and beneficence/non-maleficence, the consent must include both types of cyber risks, physical harm as well as breach of privacy/confidentiality. Thus, presenting known cyber risk information to patients will assist them in understanding: (1) their possible risks with possessing a CIED; (2) what level of risk is acceptable to them; and (3) allow them to make the best-informed decision for themselves. An example for presenting to patients the two types of cyber risks could include the following: “A cyber attack can affect you in two different ways. One is doing something to the device that affects your health. For example, a cyber attack to the device could change the settings and affect how fast or slow your heart will beat or if an electrical shock would be delivered to you when you did not need one. The other way is stealing your private medical information. This could be done, for example, from a cyber attack to the medical center, from interrupting the electronic transfer of your medical information, and/or from directly taking it from your device.” In addition, information, or guidelines on how to prevent cyber attacks, symptoms to be aware of and when to contact the provider should also be included in the informed consent [49].

Another obstacle in the inclusion of cyber risk information to the consent is how to present any and all cyber risk information as the amount and variations of cybersecurity threats are plentiful. One solution could be to provide examples of the types of cyber attacks and the potential impact to the patient in a table form in the risk section of an informed consent or as an appendix. Moreover, the abundance of cyber risk information could be overwhelming and difficult to comprehend for patients especially since providing an accurate level or percentage of risk for each type of cybersecurity threat is not feasible. An example of potential language to reflect these points in the consent could include: “Some patients may want to know what percentage of risk or what is the chance this will happen to them. We cannot say what that percentage of risk will be as cyber risks are ambiguous, meaning we cannot be exactly certain what percentage of risk you will have of a cyber attack happening to you. Also, with many cyber attacks, it is hard to find out who specifically attacked a medical center or person. This is called the attribution problem meaning we may not be able to tell you who caused the cyber attack if an attack should happen.” For good reason, patients could experience increased stress, anxiety, or distress from learning of these new potential risks to them with having a CIED. As a result, addressing the potential psychological risks for the patient in the consent would be of benefit. Such an example of this language could include, “You may find these cyber risks distressing and could cause you an increase in stress or concern. It is important to share your feelings and concerns with your provider to see if there are other care options for you or if there are mental health care options to support you should you choose to have the device implanted in you. You have the right to have these conversations with your provider at any time.” Regardless, the need to inform patients about cyber risks, despite their ambiguity and potential to overwhelm the patient, is pertinent as the patient has the right to know in order to make the best-informed decision. This could be stated, for example, as, “What we know is there are cyber risks with having an implantable device. As you are deciding if you will consent to have the device implanted in you, you have the right to know about these types of risks to you. Only you can decide if the benefits of the device are greater than the risks with having it and what number of or level of risk you will feel comfortable having.” Finally, it is important to note that the informed consent process does not end once the consent is signed but rather is an ongoing process. According to Lindsley [48], “a properly executed informed consent (IC) is a continuous process, not a singular event and thus this ‘process’ includes ongoing, interactive discussions providing patients with information sufficient to support and to maintain informed decision-making” (e.g., US Federal Regulations x45 CFR 46.116 and x21 CFR 50.20) [45,50–52]. Therefore, informing and updating the patient of new and foreseeable cyber risks, needed software updates or procedures/practices to protect them from cyber attacks and other aspects of the treatment process further supports PCC practices.

## Conclusion

Without in depth and effective communication during the informed consent process about all the cyber risks present, patients with CIEDS can be left unaware of the privacy and physical risks they possess by wearing such a device. A standardised process for medical institutions and providers to inform patients about their cyber risks is still yet to be established or finalised. Furthermore, it is a challenge to ascertain or measure the level of cyber risk as cyber attacks are often the result of: (1) an attribution problem, that is the difficulty in identifying who instigated the attack; and (2) that cyber threats are an “ambiguous threat.” While the level of risk due to a cyber attack may be small, the results could be profound to a patient and the risk of it occurring increases over time. As providers are ethically responsible for adhering to autonomy, beneficence, non-maleficence, and justice both in medical practice and the informed consent process, informing patients who have CIEDs of their risks, no matter how small, is in ethical alignment with these principles [41]. In this evolving framework of PCC, the provider has the opportunity to understand the patient’s emotional and psychological concerns with regard to possible cybersecurity risks as well as collectively discuss what is the best treatment option for that patient when factoring in all risks and benefits. “As health care is naturally more complex, financial success and health outcome success from the patient’s perspective have not been tightly coupled” [50]. Generating revenues through enhanced efficiency, which subsequently reduces the quality of informed consent and communicational practices, should not be a priority over the patient’s personal choice and right to know, which could result in the addition of unnecessary risks to the patient as well as possible negative physical and privacy outcomes. The need to educate and convey to the patient and family regarding the risk of cyber attacks with a CIED is no different.

While cyber risks with implantable medical devices have been known for over a decade, with the further advancements of AI, this need to inform patients and implement cyber-resilient practices is now of the upmost importance. “Cybercriminals have started to improve their techniques by including the Internet of Thing hacks, malware, ransomware, and Artificial Intelligence (AI), and everyone is now at risk due to the interconnectivity and intelligence of these attacks” [53]. This is especially problematic as AI technologies are evolving towards developing a learning capacity including deep learning, reinforcement learning, support vector machines, and genetic algorithms, which cybercriminals could exploit to improve the efficiency of and effectiveness in their cyber attacks on smart cyber-physical systems supported by the health care sector including using encryption itself to insert malware into the systems without being detected [53]. This type of attack would make us re-evaluate what risks would be considered foreseeable and unforeseeable and if a medical institution, provider or manufacturer could be liable for such an attack that was impossible to detect. Some would argue that now after a decade of publishing about possible cyber attacks to CIEDs, these cyber risks are truly foreseeable risks, whereas cyber attacks originating from AI could still be considered as an unforeseeable risk. Regardless, the need for ongoing improvements in data security and the safe transfer of patient data is crucial as it is only a matter of time that those with malicious intent implement a cyber attack utilising AI on patients and especially those with CIEDs. While the advancements in technology help the patient physiologically, it can hinder PCC practices if not appropriately incorporated into patient-provider dialogues. And with every step further towards technological advancement, we need to remind ourselves of keeping the human factor, the patient, at the center of it all for it is the patient in this situation who will reap the possible benefits but “carry” the burden as patients with CIEDs possess a “patient-targeted threat” with them at all times. Therefore, it is the patient’s voice and needs, whether emotional, social, medical, technological, and/or environmental, that should remain at the “center” of a choice of treatment being decided for the best possible health-effectiveness for that individual, and for being authentic to that patient’s values, beliefs, and morals. And with this increased risk and respect for their autonomy, they should be given the right to know upfront during the informed consent process.
